# Leveraging Artificial Intelligence and Modulation of Oxidative Stressors to Enhance Healthspan and Radical Longevity

**DOI:** 10.3390/biom15111501

**Published:** 2025-10-24

**Authors:** Donald D. Haines, Stephen Christopher Rose, Fred M. Cowan, Fadia F. Mahmoud, Albert A. Rizvanov, Arpad Tosaki

**Affiliations:** 1 Advanced Biotherapeutics Ltd., 20–22, Wenlock Road, London N1 7GU, UK; fadia.frcpath@gmail.com; 2 SUNY Albany College of Nanotechnology, Science, and Engineering, Albany, NY 12222, USA; chris@geronimo.life; 3 Uppsala Inc., 67 Shady Brook Drive, Colora, MD 21917, USA; fred.cowan@zoominternet.net; 4 Institute of Fundamental Medicine and Biology, Kazan (Volga Region) Federal University, 420008 Kazan, Russia; rizvanov@gmail.com; 5 Division of Medical and Biological Sciences, Tatarstan Academy of Sciences, 420111 Kazan, Russia; 6 Department Pharmacology, Faculty of Pharmacy, University of Debrecen, Nagyerdei krt. 98, 4032 Debrecen, Hungary; 7 HUN-REN-UD Pharmamodul Research Group, Faculty of Pharmacy, University of Debrecen, Nagyerdei krt. 98, 4032 Debrecen, Hungary

**Keywords:** artificial intelligence, oxidative stressors, healthspan, longevity

## Abstract

This review explores the transformative potentials of artificial intelligence (AI) in promoting healthspan and longevity. Healthspan focuses on enhancing quality of life free from chronic conditions, while longevity defines current lifespan limits within a particular species and encompasses biological aging at multiple levels. AI methodologies—including machine learning, deep learning, natural language processing, robotics, and data analytics—offer unprecedented tools to analyze complex biological data, accelerate biomarker discovery, optimize therapeutic interventions, and personalize medicine. Notably, AI has facilitated breakthroughs in identifying accurate biomarkers of biological age, developing precision medicine approaches, accelerating drug discovery, and enhancing genomic editing technologies such as CRISPR. Further, AI-based analysis of endogenous cytoprotection, especially the activity of molecules such as heme oxygenase, with particular application to hemolytic diseases. AI-driven robotics and automated monitoring systems significantly improve elderly care, lifestyle interventions, and clinical trials, demonstrating considerable potential to extend both healthspan and lifespan. However, the integration of AI into longevity research poses ethical and societal challenges, including concerns over privacy, equitable access, and broader implications of extended human lifespans. Strategic interdisciplinary collaboration, transparent AI methodologies, standardized data frameworks, and equitable policy approaches are essential to responsibly harness AI’s full potential in transforming longevity science and improving human health.

## 1. Emerging Applications of AI in Characterization of Human Aging and Countermeasures to Disease

### 1.1. Healthspan, Longevity, and Quality-of-Life

The concepts of healthspan and longevity have gained significant attention within biomedical research, driven by the ambition to not only prolong life but also enhance the quality of extended lifespans. Healthspan refers specifically to the period during which an individual maintains good health, free from chronic diseases and functional impairments associated with aging [1].

In contrast to lifespan, which merely measures the chronological length of life, healthspan emphasizes sustained physical, cognitive, and emotional well-being, underscoring the importance of quality rather than quantity alone [2]. Radical longevity, on the other hand, represents a more ambitious extension of both lifespan and healthspan, with the goal of substantially surpassing current human life expectancy limits—potentially enabling individuals to live healthy lives significantly beyond a century [3]. Radical longevity not only entails delaying age-related conditions but also actively reversing biological aging processes through targeted interventions at molecular, cellular, and systemic levels [4]. It is essential to emphasize here that at the time of this writing, radical longevity is not achievable within the limits of technology based on our understanding of the underlying mechanisms of biological aging. Thus, consideration of this concept should avoid speculative language to keep the focus on healthspan and lifespan extension grounded in current evidence.

Historically, biomedical research has focused largely on extending lifespan by reducing the incidence of life-threatening illnesses. However, contemporary geroscience—a multidisciplinary field dedicated to understanding the biological mechanisms of aging—now prioritizes improving healthspan as the critical metric of success and is substantially focused on cellular and molecular processes leading to age-dependent deterioration of cardiovascular tissue function since “death from natural causes” typically refers to various manifestations of cardiopathy [5]. To facilitate such transformative goals, researchers increasingly turn to artificial intelligence (AI), leveraging its unparalleled capability to analyze, interpret, and integrate complex biological data. AI’s inherent strengths—pattern recognition, predictive modeling, and data-driven hypothesis generation—position it as an indispensable tool for accelerating discoveries in longevity science. The present report offers hemolytic diseases as a model medical problem for AI-based analysis and design of countermeasures and addresses both the application of AI to disease treatment and modulation of aging.

These disorders provide examples of oxidative stress–driven pathologies, which in turn accelerate cardiovascular aging. While interconnected, these two paradigms are considered separately where appropriate. The use of hemolytic diseases to illustrate AI applications in solving medical problems is further considered below in Section 1.9 of this report.

### 1.2. Public Availability and Utilization of AI Platforms Needed for Simplicity of Operation

Increasingly sophisticated AI platforms available for public access hold enormous potential for accelerating the discovery of novel preventive medical and therapeutic regimens, optimized for maximum efficacy and safety, along with pricing structured to allow for appropriate use by persons in need. Moreover, these platforms are becoming increasingly user-friendly and versatile, with high potential in medical research, as well as many other human activities. A particularly exciting development in this field is the rapidly evolving integration of quantum computing into AI platforms to enhance the speed and thoroughness by which the platform may provide clear insight into problems in a wide range of fields, including but not limited to medical needs.

In developing an optimally efficient approach to the utilization of AI platforms for solving complex problems with full public access, simplification is a major priority. AI systems are designed to rapidly analyze large amounts of data pertaining to a wide range of topics. AI is capable of almost instantaneously producing multiple details of a particular problem, followed by the use of its computational power and access to current literature. A drawback to these features is that many users may feel overwhelmed by the existing operating methodology. Thus, future efforts by creators of AI products with commercial availability may increase the appeal of their products to ‘user-friendly’ user guides written to be clearly understood by literate persons with no specialized training or advanced educational certificates. The best authors for such guides will be AI systems drawing on literature describing training program design.

Literature that AI tools may access includes scientific writings and many other categories, such as engineering, law, and finance, and indeed, the entire scope of human experience is documented in various forms, all of which are readable by AI. Future AI tasks will be hugely complex and diverse. Other than medical applications, AI will be put to use in addressing social issues, trade, law enforcement, traffic, and a plethora of other socially relevant areas.

### 1.3. Integration of Quantum Computing into AI Platforms

The use of quantum computing in AI platforms will greatly amplify AI problem-solving capabilities beyond those of systems presently in use. These highly advanced computers make use of quantum mechanical principles to analyse problems and provide real-life solutions through data processing capabilities that far exceed currently available computational system capabilities. The major quantum mechanical principle underlying the enormously high computational power of this class of computer, and that sets it apart from binary computers in current use, is the utilization of “qubits”, which are the fundamental units of quantum information. Qubits are quantum analogues to classic binary bits in use by contemporary computers [5].The utilization of qubit-based systems involves the processing of data in a different manner than that of classical binary computers. Qubits exhibit a “multiple state” existence in which 1 and 0 are superimposed, facilitating a phenomenon called “quantum parallelism” in which the processing of data occurs simultaneously as opposed to the sequential nature of many classic binary computers in current use. This allows enormous data processing capabilities of all potential outcomes of, for instance, data generated in multiple experiments or regimens of clinical treatment to test a hypothesis [6].The numerous benefits to patient treatment offered as a future return on investment in quantum analysis of patient data include greatly improved targeting of specific aspects of a particular pathology and improved precision of surgical procedures resulting from quantum/AI-based analysis of medical imaging such as X-ray, ultrasound, CAT, and PET scans. Indeed, the ability of quantum-based analysis of such images to integrate fine detail with gross anatomical features thus greatly improves prevention, diagnosis, and treatment [6]. The advent of clinical and research tools that incorporate quantum-based features into AI platforms positions caregivers on the cusp of a new and very bright era in the advancement of medical science. Preliminary outcomes of the first tentative uses of this technology in patient care have already produced highly encouraging results, along with greatly enhanced insight into strategies for optimizing the computational power of AI spliced with quantum computing [7].

### 1.4. Limitations on Current AI Use

A significant caveat to current AI use is that the information must, at the moment, be archived as electronic documents, and retrieval through the internet should be possible. A major obstacle to the use of AI for analysis of information contained in academic journals is that access to many works published and available online is behind the existence of ‘paywalls’, which are cybersecurity blocks to use a particular publication without payment made to the copyright owner. Thus, at least in the near future, AI users may be compelled to either pay for single-use access to paywall-protected documents or (more practically and cost-effectively) arrange for the use of AI to screen information in academic publications through membership in or agreement with an organization such as a major university, which has a blanket agreement with copyright holders for access to a wide range of paywall-protected documents.

### 1.5. Hypothesis-Based Approach for Use of AI

Typically, an initial input parameter is entered into the AI platform in use in the form of a short summary of a particular medical problem, along with literature citations describing it. This is followed by a task assignment provided to AI, ideally in the form of a hypothesis, and four core questions posed to the AI platform in use, which generally may be phrased as follows: (1) Is the hypothesis true or false? (2) Based on the available literature scientifically validated through peer review and/or other screening for truthful content and archived as electronic documents accessible and readable in public domain through the AI platform in use (here, the name of the platform is inserted), and accessible through the Internet, (3) what evidence may be used to conclude that the hypothesis is true, and (4) what evidence may be used to conclude that the hypothesis is false?

### 1.6. Representative Strategy for Using an AI Platform to Solve a Complex Problem

The following three task assignments are given to the AI platform in use:

Subtask assignment 1. for AI: Using reference literature fitting the criteria (1)–(4) above and including content on methodology used to answer research questions pertaining to the hypothesis to be tested (as stated above), design experiments by which researchers may test the hypothesis.

Subtask assignment 2. for AI: For each statement made by either the AI platform or a human author, which derives from previously published literature, cite the source and place it in the main text and at the end of the manuscript, using the reference formatting style required by the journal.

Subtask assignment 3. for AI: Using reference literature fitting the criteria (1)–(4) above and including content on methodology used to answer research questions pertaining to the hypothesis to be tested (as stated above), design experiments by which researchers may test the hypothesis.

The foregoing investigative framework provides a format by which a particular problem related to a medical need may be presented to an AI platform, phrased in such a manner that the platform may provide an answer to the investigators useful in solving the problem.

The protocol for analyzing the medical problem will involve first providing the AI platform with a description of the problem, along with suggested hypotheses for its solution. This information is the major input parameter to AI for a particular mission assignment. Additional mission-specific subtasks will be given to the AI tool to enable it to electronically access all the background information, so a cost-effective, rapid, experimental test of the most promising hypothesis may be designed.

### 1.7. Oxidative Stress, “Inflammaging”, and Antioxidant Countermeasures

As AI-based analysis of medical needs evolves, its problem-solving capabilities will increasingly be focused on a major hallmark of the progressive deterioration of healthy organ function over the lifespan of an individual. This is an age-dependent increase in inflammatory oxidative stressors produced by components of the immune system and also by senescent cells, which are non-proliferative cell types typically arising from the destabilization of the genome due to cell injury or erosion of telomeres in cells that have reached a maximum number of doublings—called the Hayflick Limit [8]. This phenomenon, called “inflammaging”, is elegantly described by Nicole Ehrhart, V.M.D., director of Colorado State University’s Columbine Health Systems Center for Healthy Aging, saying: “As we age, our immune system gets less specific to what it’s reacting to. Over time, this cumulative chronic response becomes this low-grade chronic inflammation” says Ehrhart. “It doesn’t’ function as it used to when we were younger”. “The result is inflammaging” [9]. Lifestyle influences that trigger or exacerbate inflammaging by promoting increasingly prooxidant environments in critical tissues such as cardiovascular, kidney, and neural cells include factors such as chronic infections with associated inflammation, and toxicant exposure [10], resulting in chronic inflammation with accelerated aging. Obesity is also a lifestyle-associated contributor to increasingly prooxidant tissue environments and consequent age-associated physical decline since adipocytes express high levels of inflammatory mediators, perpetuating a vicious cycle that disrupts the regulation of inflammatory processes, in turn leading to features of metabolic disorder such as insulin resistance and dyslipidemia, which increase in incidence with age [11]. Encouragingly, inflammaging and the resulting accelerated aging may be inhibited by various plant polyphenols [10].It is thus reasonable to predict that in the near future, AI platforms may be capable of designing experiments using input parameters of properties of phytochemical compound libraries and designing in vitro experiments that test the full contents of the libraries using automated cell culture or cell-free systems. For these experiments, the AI tool will design evaluations of combinations of compound library contents using models with outcome variables predictive of age-associated biomarker expression for selected pathologies. For example, in 2013, Angelique Camilleri et al. at the University of Malta demonstrated the capacity of selected plant polyphenol combinations to protect mitochondrial membranes from permeabilization by amyloid aggregates, which is an accepted in vitro model for the development of countermeasures to Alzheimer’s disease [12]. The University of Malta investigators themselves planned the testing matrices using their own reasoning and produced results of clinical value to ongoing progress in the management of Alzheimer’s, Parkinson’s, and other neurological disorders. An advanced extension of their methodology would require an appropriately configured AI tool that would enable their experimental paradigm to be vastly scaled up. Nevertheless, a limitation on the number of combinations of components of a substance library that might be tested for the effect on the magnitude of expression of biological markers of aging or age-associated disease would be the requirement for a “wet” phase in the investigation, where substance combinations are added to cell cultures or cell-free models such as the mitochondrial membranes used by Camilleri et al. [12]. Currently, this obstacle imposes time- and resource-related constraints on the scale and speed with which combinations of contents of a substance library may be cross-evaluated. There is, nevertheless, some cause for optimism that this may be surmounted using “virtual cell” models in which AI builds a virtual replica of a cell or component thereof (such as a mitochondrion), configured to the engagement of virtual membranes and receptors constructed to relay signals from single molecules or mixtures [13]. For such a screening system to be practical, components of the virtual cell, particularly membranes and receptors that interact with external stimuli, would need to be well-characterized to provide input data for the AI tool designing the cell. At the time of this writing, there is a great deal of information available on all known components of cells from many species. However, many gaps exist and it may be years before virtual cells can be created that faithfully replicate responses of living tissue—particularly for experiments using combinations of phytochemicals and other substances with compositions that may include molecules for which biological targets have not been fully defined (which is necessary for any component of a virtual cell or virtual compounds that may interact with it). These factors notwithstanding, AI tools, particularly quantum-based systems, have the intrinsic data-processing power to simulate cells and components thereof to produce data of value in solving problems in a wide range of fields—including ongoing efforts to extend healthspan by minimizing the adverse impact of inflammaging [14].

### 1.8. Inflammatory Damage to Cardiovascular Tissue, Death from “Old Age”, and Progeria

As an individual ages, regulatory mechanisms that constrain inflammation to its appropriate physiologic venues become progressively less efficient, resulting in a wide range of chronic diseases [15]. Some tissues undergo age-associated functional impairment at a higher rate than others. For example, cells of the cardiovascular system are highly sensitive to oxidative damage of the kind imposed by inflammaging. Thus, death from “old age” is typically the result of impaired function of cardiovascular tissue [15]. Moreover, cells of individuals with Progeria, a disorder involving a genetic defect that results in the emergence of a high burden of pro-inflammatory senescent cells, typically die of heart failure [15].

### 1.9. Hemolytic Diseases: A Representative Research Challenge for AI

Hemolytic diseases are typically characterized by an underlying pathogenesis driven by the reactivity of free heme released from hemoglobin in red blood cells during normal physiologic activity, such as red blood cell turnover, or syndromes in which pathological hemolysis occurs. These include syndromes such as Hemolytic anemia, Hemolytic–uremic syndrome, and related disorders [16,17]. The pathogenesis of hemolytic disorders is triggered and driven by the capacity of unreacted heme to damage vascular endothelium by catalyzing expression of high levels of reactive oxygen species (ROS), along with pro-inflammatory transcription factors such as AP-1 and NF-κB, along with pro-inflammatory cytokines [18] and inappropriate complement activation [19]. These effects exacerbate pathological oxidative stress with resulting inflammatory tissue damage and also contribute to the rate and severity of inflammaging. Physiologic countermeasures to the vasculotoxic effects of hemolysis include heme clearance by the hemopexin/CD91 and haptoglobin/CD163 systems, respectively, and notably, heme oxygenase, which degrades heme to bioactive products of heme degradation that include carbon monoxide (CO) and biliverdin/bilirubin, which are strongly cytoprotective, as well as with ferrous iron, a potentially toxic molecule that is sequestered by ferritin, an intracellular iron-storage protein that releases iron in a controlled manner [18]. These metabolites may either exacerbate or ameliorate inflammaging depending on the redox microenvironment of the affected tissue and the pathogenic processes acting on it [20]. The authors of the present report have demonstrated in multiple in vitro, animal, and human trials that a potent heme oxygenase inducer derived from seeds of sour cherry (*Prunus cerasus*) dramatically abated symptoms of osteoarthritis [21], with clear application to all diseases exhibiting oxidative damage to affected tissues as a result of dysregulated inflammatory processes [21,22]. The sour cherry seed extracts used in the above-mentioned research are generally regarded as safe (GRAS) natural medical material (NMM) and were shown by the investigators to be non-toxic in a rodent model, even at levels 200 times the therapeutic dosage [23]. Additionally, the use of phytochemicals in medical therapies [24] has a substantial benefit for the prevention of cardiac disorders in connection with heme oxygenase activities and ventricular arrhythmias [25,26].

### 1.10. Strategies for Use of AI in Design of Research on Hemolytic and Other Diseases

Here, hemolytic diseases are presented as disorders with symptoms that correlate positively with age. It is important to emphasize that the use of AI described below to optimize the treatment of hemolytic anemia in dogs is exemplary, not limiting, and is applicable to the use of AI to develop optimal solutions to any problem. Within the scope of the ongoing characterization of the aging process and the development of countermeasures, with use over time, it is inevitable that the natural curiosity and innovative talent of participants in this quest will create a huge and growing body of AI methodology focused on extending the human lifespan. This is very exciting and may prove to be a revolutionary milestone in the evolution of the human condition.

Sample strategy for AI use: AI will be used to propose experimental and clinical protocols to test a hypothesis that a bioactive agent, here named AFFECTOR-N1, will mitigate symptoms of hemolytic anemia by decreasing rates and extent of pathological lysis of red blood cells. In the broad investigative paradigm, AFFECTOR-N1 may be a drug, supplement, surgery, acupuncture, or phototherapy—or any of a diverse range of interventions. For the present example of how the hypothesis-based approach for the use of AI (described above) may be used, AFFECTOR N1 is a commonly used orally delivered pharmaceutical called the Immune-Mediated Hemolytic Anemia (IMHA) Package, used to treat hemolytic anemia in dogs. It is manufactured and marketed by Pet Health and Nutrition Center in New Hartford CT, USA.06057.

Major hypothesis given to AI: AFFECTOR-N1 (IMHA) when combined with AFFECTOR N2 (placebo) will show improved prognoses of hemolytic anemia symptoms in dogs.

Subtask (1): assignment for AI: Access all published literature describing the use of a canine model to evaluate drugs and design a randomized double-blind placebo–control study to test the foregoing hypothesis. The foregoing investigative framework provides a format by which a particular problem related to a medical need may be presented to an AI platform, phrased in such a manner that the platform may provide an answer to the investigators useful in solving the problem. As an individual ages, there is a progressive decline in the ability of an individual to clear unreacted heme through mechanisms such as heme oxygenase-mediated degradation of the molecule, resulting in a plethora of medical problems and significantly worse quality of life [18].

### 1.11. Integration of and Distinctions Between Medical Treatments and Healthspan Enhancement

**Figure 1:**
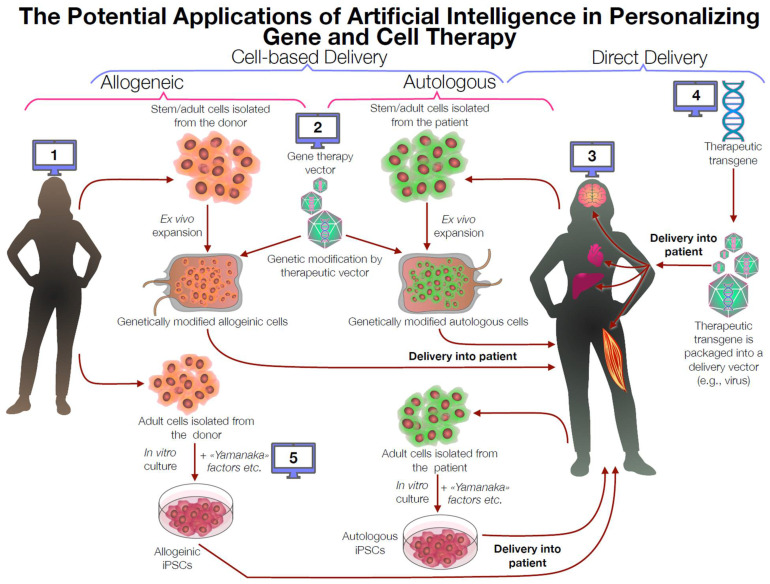
The Potential applications of artificial intelligence in personalizing gene and cell therapy. To enhance healthspan and promote radical longevity, gene and cell therapies aim to target organism-level systems, specific organs/tissues, and cellular processes that drive aging and age-related decline. Organismal systems: brain (CNS): prevent neurodegeneration (Alzheimer’s, Parkinson’s); musculoskeletal: prevent sarcopenia, osteoporosis; immune system: reverse immunosenescence, inflammaging; cardiovascular: prevent atherosclerosis, fibrosis; liver: treat age-related metabolic dysfunction; skin: reverse skin aging, enhance wound healing; systemic/whole body: multi-organ rejuvenation. Cellular processes: Genomic Instability, Telomere Attrition, Epigenetic Alterations, Loss of Proteostasis, Mitochondrial Dysfunction, Cellular Senescence, Stem Cell Exhaustion, Altered Intercellular Communication.

The major purpose of this report is to offer basic and clinical researchers and healthcare providers an easily read overview of ways in which AI may be used to augment their primary missions. This article discusses aspects of both disease treatment and approaches to the optimization of healthy aging, two interconnected concepts that are addressed in integrated form, but with distinct applications to clinical and/or research relevance provided. A ‘snapshot’ example of how these two concepts are combined while underscoring a distinction between treatment and healthspan enhancement is shown in Figure 1, showing a clear pictorial representation of AI application to personalized medicine.

## 2. AI Capabilities and Applications in the Development of Research Strategies for Healthy Aging

### 2.1. Brief Overview of AI Capabilities

Artificial intelligence (AI) encompasses an array of computational methodologies designed to mimic or augment human cognitive functions, including perception, reasoning, decision-making, and learning. At its core, AI leverages advanced algorithms and computational power to interpret complex data, uncover hidden patterns, and predict outcomes with unprecedented precision and speed [27]. Within longevity research, the primary AI methodologies currently employed include machine learning (ML), deep learning (DL), natural language processing (NLP), robotics, and sophisticated data analytics. Machine learning (ML) refers to algorithms that enable computational systems to iteratively “learn” from data, refining their performance through experience without explicit programming. ML includes supervised learning—where models train on labeled datasets—and unsupervised learning—where algorithms discern inherent patterns within unlabeled data [28]. Such capabilities are especially critical in longevity science for biomarker discovery, predicting biological age, and optimizing patient-specific therapies. Deep learning (DL), a specialized subset of ML, uses neural network architectures modeled loosely on the human brain’s interconnected neurons. DL excels at extracting intricate features and relationships from vast, high-dimensional datasets—such as genomic sequences, medical imaging, and multi-omics biological data—which often elude traditional analytical methods [29]. The ability to process complex biological data rapidly positions DL algorithms as powerful tools for uncovering previously unknown aging-related molecular mechanisms. Natural language processing (NLP), another AI technique, systematically interprets and extracts meaningful information from unstructured textual data. In longevity research, NLP applications are increasingly used to mine vast repositories of scientific literature, clinical notes, and electronic health records to inform clinical decision-making, facilitate knowledge discovery, and accelerate hypothesis generation and testing [30]. Furthermore, robotics and automation technologies are often integrated with AI-supported elderly care and health monitoring. Robotics equipped with AI-driven predictive analytics assist in detecting early signs of health deterioration, enhance independent living capabilities, and offer personalized assistance in daily activities [31]. Automation similarly aids precision monitoring and adaptive interventions designed to improve healthspan outcomes. Lastly, advanced AI-driven data analytics and modeling significantly enhance the efficiency and effectiveness of clinical trials and longevity studies by enabling optimized trial designs, precise participant stratification, and dynamic, real-time outcome tracking [32]. In summary, the diverse capabilities of AI offer revolutionary tools to accelerate and amplify research in healthspan extension and radical longevity, serving as catalysts for transformative scientific advancements.

### 2.2. Significance of Integrating AI into Longevity Research

The integration of artificial intelligence into longevity research marks a pivotal advancement, enabling researchers to address fundamental challenges that traditional methodologies have struggled to overcome. AI’s capability to rapidly process vast, heterogeneous biological datasets provides researchers unprecedented opportunities to unravel the complexity of aging mechanisms, accelerating the discovery of biomarkers, therapeutics, and personalized interventions aimed at increasing healthspan and radical longevity. Aging, at its core, is a highly intricate biological phenomenon, involving interactions at multiple levels—genomic, proteomic, metabolomic, and environmental. Traditional experimental and analytical approaches have often been insufficient to fully comprehend or effectively intervene in such a multifaceted process [33]. In contrast, AI-driven analytics can systematically identify hidden relationships within large-scale datasets, uncover novel hypotheses, and facilitate precise, predictive modeling of aging dynamics [34]. One particularly impactful advantage of integrating AI into longevity research is its potential to identify biomarkers of aging efficiently and reliably. Accurate biomarkers are essential for diagnosing biological age, assessing intervention efficacy, and guiding personalized strategies for healthspan extension [35]. Furthermore, AI accelerates the traditionally slow and costly process of drug discovery, offering virtual screening, predictive simulations, and even drug repurposing opportunities specifically tailored toward targeting aging-related pathways [33,36]. AI’s capacity for personalized prediction and intervention also transforms longevity medicine into individualized monitoring—promising more effective outcomes by tailoring interventions to specific genetic backgrounds, physiological states, and environmental factors unique to everyone [37]. Moreover, AI significantly enhances the efficiency and outcomes of clinical trials and longevity studies. Through improved participant selection, real-time monitoring, adaptive trial designs, and optimized resource allocation, AI-driven approaches can markedly reduce costs and accelerate the translation of experimental findings into clinical practice [32]. Lastly, the integration of robotics and AI automation holds promise for dramatically improving elderly care, not only through enhanced daily assistance and proactive health interventions but also by alleviating pressures on healthcare systems increasingly burdened by aging populations [31]. In summary, embedding AI within longevity research substantially amplifies researchers’ abilities to understand, intervene in, and ultimately transform aging processes. Leveraging AI not only accelerates scientific discovery but also paves the way toward realizing the ambitious goal of significantly extending healthspan and achieving radical longevity.

### 2.3. AI in Biomarker Discovery and Aging Diagnostics

#### 2.3.1. Machine Learning and Deep Learning in Identifying Aging Biomarkers

Identifying reliable biomarkers that accurately reflect biological age rather than chronological age is essential for longevity science. Traditional approaches in biomarker discovery often involve isolated biological measures, typically limited by low sensitivity, specificity, or predictive power [35]. By contrast, machine learning (ML) and deep learning (DL) methodologies significantly advance the capacity to discover, validate, and utilize biomarkers predictive of aging processes and healthspan. Machine learning algorithms—particularly supervised learning techniques like Random Forests, Support Vector Machines (SVMs), and Gradient Boosting—have proven effective in systematically identifying aging biomarkers within complex biological datasets, such as gene expression profiles, metabolomics, and proteomics [38]. Deep learning methods, particularly convolutional neural networks (CNNs) and recurrent neural networks (RNNs), further expand this capability by modeling nonlinear, high-dimensional biological relationships, thereby uncovering subtle molecular signatures that more accurately reflect biological aging [39]. AI approaches, especially DL, excel at integrating diverse multi-omics data sources (e.g., genomic, transcriptomic, epigenomic, proteomic, and microbiomic datasets), facilitating a comprehensive understanding of aging’s multifaceted nature. For instance, DL has enabled the identification of intricate epigenetic changes and transcriptomic signatures associated with biological aging, contributing significantly to the development of the well-known epigenetic “aging clocks” [40].

#### 2.3.2. AI-Driven Diagnostic Tools Predicting Biological Age

AI-driven diagnostic tools have emerged as powerful instruments for accurately determining biological age, thereby transforming diagnostics in longevity medicine. These tools leverage AI’s capacity to integrate and analyze multi-dimensional biomarker data, providing predictive analytics for aging trajectories, disease risk assessment, and healthspan optimization [41]. Several AI-derived biological age predictors—such as DNA methylation clocks (epigenetic clocks), transcriptomic age estimators, and imaging-based aging biomarkers—demonstrate remarkably accurate predictive power. These diagnostic tools not only facilitate early detection of aging-related health deterioration but also enable precise tracking of intervention outcomes, guiding individualized longevity interventions [42]. AI algorithms are instrumental in extracting aging-related patterns from medical imaging data, such as MRI scans or retinal images, offering non-invasive approaches for biological age determination. For example, DL algorithms applied to retinal imaging data have successfully predicted cardiovascular risk factors and biological age with high accuracy, thus opening novel pathways for aging diagnostics [43].

#### 2.3.3. Case Studies of Successful Biomarker Identification Using AI

Recent literature has provided numerous examples demonstrating the utility and success of AI in aging biomarker discovery. One landmark study developed a deep neural network capable of accurately predicting biological age and associated mortality risks based solely on blood biochemical parameters, significantly outperforming traditional statistical methods [44]. This study illustrated the potential of AI to identify biomarkers that may not have been previously recognized as relevant to aging processes. Another influential case study involves the epigenetic clock, pioneered by Horvath, which uses machine learning algorithms to interpret DNA methylation patterns as robust biomarkers for biological aging [40]. Subsequent refinements of this methodology, driven by increasingly sophisticated AI techniques, have further enhanced its accuracy, predictive value, and clinical applicability [42]. Similarly, AI-driven analyses of transcriptomic and metabolomic data have identified biomarkers capable of predicting biological age, lifespan, and healthspan in animal models and human populations, demonstrating the profound predictive and diagnostic utility of machine learning approaches in biomarker discovery [38,45]. These successes underscore AI’s profound potential to redefine aging diagnostics, providing both robust biomarkers and practical clinical tools critical for advancing healthspan and radical longevity research.

### 2.4. AI in Drug Discovery and Therapeutics

#### 2.4.1. Accelerated Drug Discovery Processes

The drug discovery process, traditionally lengthy, expensive, and characterized by high failure rates, has been significantly accelerated through the application of artificial intelligence (AI). Conventional pharmaceutical development can span more than a decade from initial target identification to clinical approval, with costs often exceeding billions of dollars per successfully marketed drug [46]. AI methodologies—especially machine learning (ML) and deep learning (DL)—have transformed this paradigm, drastically reducing timeframes and costs associated with identifying therapeutic candidates for longevity applications [47]. AI-driven platforms systematically analyze extensive molecular, genomic, and clinical databases to rapidly identify and prioritize promising molecular targets and drug candidates. AI systems such as generative adversarial networks (GANs) and reinforcement learning algorithms can efficiently predict molecular interactions, optimize chemical structures, and virtually screen enormous libraries of known and novel compounds [48]. By identifying viable drug candidates and excluding suboptimal compounds at early stages, AI substantially accelerates preclinical development and reduces attrition rates associated with ineffective or toxic molecules.

#### 2.4.2. AI-Driven Simulation and Drug Efficacy Modeling

AI-driven computational modeling represents another transformative aspect of accelerated drug discovery, especially in longevity research. Virtual simulations empowered by AI techniques, including molecular dynamics simulations and in silico pharmacodynamics modeling, enable precise predictions of drug efficacy, pharmacokinetics, and potential side effects without extensive laboratory or animal testing [49]. Deep learning models have been particularly effective in predicting interactions between drugs and aging-associated molecular pathways. For instance, AI-driven simulations can model the effects of candidate molecules on cellular senescence, mitochondrial function, autophagy pathways, and epigenetic modifications, significantly accelerating identification of compounds with longevity-enhancing properties [47]. Such simulations can identify candidates likely to produce favorable therapeutic outcomes, enabling researchers to prioritize the most promising molecules for further development.

#### 2.4.3. Personalized Medicine Using AI Analytics

AI has significantly enhanced personalized medicine strategies within longevity therapeutics by enabling tailored interventions based on individual biological profiles. By analyzing vast arrays of personalized data—including genomic, transcriptomic, proteomic, and phenotypic information—AI models can accurately predict individual responses to longevity therapeutics, optimizing treatment selection and dosage to maximize efficacy and minimize adverse reactions [50]. Advanced AI analytics facilitate precision longevity interventions by predicting how individual genetic backgrounds influence drug metabolism, responsiveness, and long-term therapeutic outcomes. For example, AI models integrating genetic information with clinical biomarkers have successfully guided the personalized administration of senolytic drugs, mitochondrial enhancers, and metabolic modulators, dramatically enhancing individual healthspan outcomes [51].

#### 2.4.4. Examples of Successful AI-Accelerated Longevity Therapeutics

Several notable examples illustrate the successful application of AI in accelerating drug discovery and therapeutics development within longevity medicine. One landmark example is the identification of novel senolytic compounds—agents capable of selectively eliminating senescent cells implicated in aging—through AI-driven drug screening and generative modeling. Such AI-based approaches enabled researchers to discover entirely new chemical entities with superior efficacy and reduced toxicity profiles compared to traditionally developed senolytics [52]. Another prominent example involves AI-assisted repurposing of existing drugs for longevity applications. AI algorithms successfully identified rapamycin analogs and metformin derivatives that demonstrate potential lifespan and healthspan-extending effects through targeted modulation of metabolic and cellular pathways [36]. AI-guided discovery and validation of NAD+ boosters and mitochondrial-targeted therapeutics represent additional cases where AI analytics significantly expedited the progression from discovery to clinical evaluation, underscoring the transformative impact of AI on longevity therapeutics [53]. Overall, the integration of AI into drug discovery and therapeutics for longevity research has proven pivotal in accelerating the identification, validation, and optimization of interventions aimed at extending healthspan and promoting radical longevity.

### 2.5. AI and Genomics in Longevity Research

#### 2.5.1. Analysis and Interpretation of Genomic Data Using AI

Genomic data plays a pivotal role in longevity research, providing insights into genetic determinants of aging, lifespan variability, and age-related disease susceptibility. However, interpreting genomic data is inherently complex due to its high dimensionality, volume, and the multifactorial nature of genetic influences on aging. Artificial intelligence, particularly through machine learning (ML) and deep learning (DL) methodologies, significantly enhances genomic data analysis by efficiently capturing complex gene–gene and gene–environment interactions influencing aging processes [54]. Machine learning algorithms such as Random Forests, Support Vector Machines (SVMs), and gradient boosting methods effectively identify genetic variants associated with longevity phenotypes within large-scale genomic datasets, including genome-wide association studies (GWAS) and whole-genome sequencing (WGS) data [38]. Deep learning techniques, particularly convolutional neural networks (CNNs), have proven powerful in identifying regulatory elements, interpreting gene expression patterns, and predicting functional impacts of genetic variants associated with aging and longevity [55]. AI-driven computational frameworks, such as integrated multi-omics analysis platforms, further enhance the interpretation of genomic data by combining genomic information with transcriptomic, epigenomic, and proteomic datasets. These integrative approaches facilitate identification of robust genetic markers for biological aging, predictive disease-risk profiling, and targeted therapeutic strategies [56].

#### 2.5.2. AI-Powered Gene Editing Technologies

AI technologies significantly enhance gene-editing techniques, particularly CRISPR-based systems, by increasing precision, efficiency, and predictability—key attributes necessary for applying gene editing to longevity research. CRISPR technologies enable targeted genetic interventions capable of directly modulating aging-related genetic pathways, potentially reversing deleterious aging processes at the cellular and organismal levels [57]. Artificial intelligence supports gene editing by predicting optimal target sites, minimizing off-target effects, and enhancing editing accuracy and efficiency. AI-powered algorithms, particularly deep learning models, systematically evaluate genomic sequences to predict editing efficiency, off-target impacts, and functional outcomes of gene-editing interventions, dramatically improving therapeutic efficacy and safety profiles [58]. Such predictive models are essential for designing genetic interventions targeting critical longevity-associated pathways, including cellular senescence, telomere length maintenance, DNA repair mechanisms, and metabolic regulation [59].

#### 2.5.3. Predictive Genomics for Personalized Longevity Strategies

AI-driven predictive genomics is a cornerstone of personalized longevity interventions, facilitating precise, individualized predictions of aging trajectories, disease susceptibilities, and optimal intervention strategies. By integrating genomic data with environmental, clinical, and lifestyle information, predictive genomic models—empowered by AI—offer unprecedented accuracy in tailoring personalized longevity strategies [60]. Machine learning and deep learning models can predict individual susceptibility to age-related diseases, responsiveness to dietary or pharmacological interventions, and optimal preventive strategies based on individual genetic profiles. For instance, AI-driven genomic analyses have demonstrated effectiveness in predicting individual risk and responsiveness to interventions targeting metabolic aging, cardiovascular aging, neurodegeneration, and inflammation, thus informing personalized preventive and therapeutic longevity interventions [61]. Additionally, polygenic risk scores (PRS), refined through AI analytics, have significantly improved personalized longevity predictions. AI-enhanced PRS incorporates thousands of genomic variants into comprehensive risk profiles, providing powerful tools for preventive medicine by enabling early, individualized interventions aimed at extending healthspan and lifespan [62]. In summary, integrating AI with genomics dramatically accelerates the discovery, interpretation, and clinical application of genetic insights into aging and longevity, substantially advancing personalized, precision-focused longevity strategies.

#### 2.5.4. AI-Driven Design and Personalization of Gene and Cell Therapies

The integration of artificial intelligence (AI) with gene and cell therapy represents a revolutionary approach in longevity research, enabling precise and personalized interventions at the genetic and cellular levels aimed at significantly extending healthspan and achieving radical longevity. Gene and cell therapies have the potential to directly modify or repair genetic and cellular processes associated with aging, including genomic instability, cellular senescence, mitochondrial dysfunction, and stem cell exhaustion [63]. However, these therapies are inherently complex, requiring intricate manipulation of biological systems to achieve both efficacy and safety. AI methodologies, particularly deep learning (DL) and machine learning (ML), greatly enhance the development, precision, and effectiveness of these therapeutic strategies [64]. AI is being used to design gene therapies by optimizing vectors, such as creating AAV capsids that evade immunity and target specific tissues, and generating diverse, viable variants that can be used in the context of anti-aging therapy [65]. Additionally, AI can design new gRNA sequences for CRISPR-based genome editing, streamlining the process for clinical-grade therapies targeting age-related diseases [66]. AI plays a critical role in optimizing cell therapies, particularly those involving stem cells and induced pluripotent stem cells (iPSCs) [67,68]. AI-driven analyses have significantly accelerated the development of genetically engineered mesenchymal stem cells capable of reducing inflammation and enhancing regenerative capacities, thus mitigating age-related decline. Bioinformatic analysis, supported by AI of gene expression data, helped identify uPAR as an ideal target for senescent cells, and ongoing machine learning research is exploring other senescence markers that CAR-T cells or antibodies might target [69,70]. In the future, AI may guide complex regenerative procedures, such as in vivo reprogramming, and minimize malignant transformation during partial reprogramming, for example, by using Yamanaka OSK factor genes.

### 2.6. AI-Enhanced Clinical Trials and Longevity Studies

#### 2.6.1. AI in Patient Recruitment, Monitoring, and Analysis

Effective patient recruitment, monitoring, and analysis represent critical bottlenecks in clinical research, particularly within longevity studies, which often span extended timeframes and demand precise participant selection to accurately assess interventions aimed at healthspan extension. Artificial intelligence (AI), including machine learning (ML) and natural language processing (NLP), offers innovative solutions to address these challenges, substantially enhancing trial efficiency, accuracy, and success rates [32]. AI-driven algorithms streamline patient recruitment by analyzing electronic health records (EHRs), clinical databases, and patient registries, quickly identifying and stratifying suitable participants based on detailed inclusion and exclusion criteria. NLP techniques enable rapid extraction and synthesis of patient eligibility criteria from complex medical records, dramatically reducing recruitment timelines and improving participant suitability for longevity interventions [71]. Furthermore, AI technologies enhance patient monitoring through wearable sensors, mobile health applications, and telemedicine platforms, capturing real-time, continuous physiological, behavioral, and biometric data. ML models analyze this streaming data to detect subtle physiological shifts or adverse responses, enabling proactive, personalized intervention adjustments that enhance trial safety and improve data quality [72].

AI methodologies play a transformative role in optimizing clinical trial design, significantly reducing the traditionally high costs and lengthy durations of clinical research—particularly relevant in longevity studies where trials may extend over many years. Machine learning and predictive analytics enable more precise trial designs, including adaptive designs, to rapidly and efficiently identify optimal dosing, duration, and patient subpopulations [73]. AI-driven predictive modeling supports adaptive clinical trials by dynamically adjusting trial parameters in response to interim data analyses. This capability enhances decision-making regarding patient cohorts, endpoints, and intervention strategies, reducing unnecessary exposure to ineffective treatments and optimizing the allocation of research resources toward the most promising longevity interventions [74]. Advanced AI techniques also facilitate realistic simulation of clinical trials prior to execution, helping researchers anticipate potential outcomes, risks, and resource needs. Such AI-based simulation studies significantly enhance trial planning, particularly in complex, long-term studies focused on aging interventions, ensuring robust and efficient study designs [32].

#### 2.6.2. Real-Time Data Analytics for Efficient Longevity Research Outcomes

The application of AI-powered real-time data analytics represents another crucial innovation in longevity research, enabling rapid, precise, and data-driven decisions that improve clinical trial outcomes. Real-time analytics powered by AI allows immediate evaluation of trial data, facilitating early identification of significant trends, adverse events, or unexpected outcomes that can dramatically influence trial progression. Machine learning models analyzing real-time biomarker, clinical, and physiological data can immediately identify participants responding positively or negatively to interventions, enabling precise adjustments or personalized modifications in real-time. Such real-time predictive analytics significantly enhance the capacity of longevity researchers to detect subtle, biologically relevant intervention effects, reducing delays and resource waste associated with traditional retrospective analyses [72]. Additionally, AI-enabled platforms can automatically synthesize and interpret real-time data from multiple trial sites, ensuring consistency, accuracy, and immediate actionable insights. This real-time integration capability is particularly valuable in large-scale, multi-center longevity trials, allowing researchers to rapidly respond to emerging trends, maximize trial efficacy, and enhance overall data reliability and research outcomes [32]. In summary, integrating AI into clinical trials and longevity studies significantly accelerates research processes, improves trial design efficiency, optimizes resource utilization, and enhances the precision and applicability of clinical outcomes essential for translating longevity science into practice.

### 2.7. Robotics, Automation, and AI in Elderly Care

#### 2.7.1. AI-Enhanced Robotics Assisting Elderly Populations

As global populations age, the demand for effective elderly care has grown significantly, posing substantial challenges for healthcare systems worldwide. Robotics enhanced with artificial intelligence (AI) represents a transformative solution capable of improving care quality, safety, and independence among elderly individuals [32]. AI-powered robotic systems can assist older adults in daily activities, mobility support, medication reminders, and emotional companionship. Robots embedded with advanced machine learning capabilities and natural language processing facilitate intuitive, responsive interactions tailored to individual preferences and physical or cognitive needs. For example, socially assistive robots such as PARO and Jibo have demonstrated effectiveness in reducing loneliness, enhancing emotional well-being, and supporting cognitive engagement among elderly users [75,76]. Furthermore, AI-driven robotic mobility aids and exoskeletons significantly enhance independence by assisting elderly individuals in tasks such as walking, transferring between postures, and performing physical rehabilitation. These intelligent robotic devices adapt to each user’s abilities and changing physiological conditions, thereby contributing substantially to prolonged autonomy, reduced fall risk, and improved overall quality of life [77].

#### 2.7.2. Automation in Monitoring and Proactive Health Interventions

AI-driven automation in elderly care extends beyond robotics, encompassing sophisticated monitoring systems that proactively assess health status and predict emerging health risks. Such systems integrate continuous data from wearable devices, smart-home sensors, and remote health-monitoring technologies, employing predictive analytics to identify early signs of health deterioration, enabling timely intervention [78]. Machine learning algorithms continuously analyze real-time physiological parameters such as heart rate variability, gait patterns, sleep quality, and medication adherence, identifying subtle deviations that signal potential health risks. Automated alerts triggered by AI-based predictive analytics facilitate prompt healthcare provider interventions, reducing emergency situations, hospital admissions, and complications associated with chronic age-related conditions [79]. AI-driven home automation and ambient assisted living environments integrate these predictive analytics into smart-home frameworks, further enabling proactive interventions. Such systems can automatically adjust environmental conditions to optimize comfort, safety, and health outcomes—for example, adaptive lighting systems to prevent falls, automated medication dispensing to enhance compliance, or voice-activated emergency assistance activated when predictive analytics detect imminent health risks [79].

#### 2.7.3. Ethical Implications and Challenges in Robotic Elderly Care

While robotics and automation offer profound benefits for elderly care, the integration of AI-driven systems raises significant ethical, social, and practical challenges. Issues surrounding privacy, autonomy, consent, and emotional dependency must be carefully addressed to ensure ethical adoption and sustained acceptance by elderly populations and their caregivers [80]. AI-enhanced robotic caregiving raises concerns regarding the potential for reduced human contact, social isolation, and emotional reliance on robots. Researchers and healthcare providers must carefully balance robotic assistance with sufficient human interaction, ensuring that robotic interventions complement rather than substitute meaningful human caregiving relationships [81]. Privacy and data security also represent critical ethical considerations. Continuous monitoring and data collection by AI-driven systems necessitate robust privacy protocols and data governance policies to safeguard sensitive personal information and maintain trust among elderly users and caregivers [79]. Additionally, equitable access remains a significant challenge. Advanced robotic caregiving technologies risk widening socio-economic disparities if high costs or technical complexity limit accessibility to affluent or tech-savvy populations. Strategic initiatives are required to ensure the inclusive, equitable distribution of these transformative caregiving technologies [80]. In conclusion, AI-enhanced robotics and automation offer transformative potential for elderly care, enhancing safety, independence, and health outcomes. However, successful integration requires careful attention to ethical, social, and practical considerations, ensuring that these technologies serve as supportive complements to humane caregiving.

### 2.8. AI for Lifestyle and Behavioral Intervention

#### 2.8.1. Personalized Lifestyle Recommendations Driven by AI

Lifestyle factors such as diet, physical activity, sleep, and stress management significantly influence healthspan and longevity. Artificial intelligence (AI) has become increasingly integral in developing personalized lifestyle recommendations, tailoring interventions to an individual’s unique genetic makeup, physiological conditions, environmental exposures, and behavioral patterns [82]. AI-driven predictive algorithms analyze comprehensive datasets derived from genomics, wearable sensors, electronic health records (EHRs), and lifestyle questionnaires. Machine learning models can accurately identify individual risks and opportunities related to metabolic health, cardiovascular function, cognitive resilience, and overall aging trajectories [34] These personalized predictions facilitate the development of precise and effective lifestyle interventions, optimizing behaviors such as nutritional intake, exercise regimens, sleep routines, and stress reduction strategies [82,83]. For instance, AI-based nutritional platforms generate individualized dietary plans by analyzing personal biomarkers, microbiome profiles, and metabolic responses, significantly improving metabolic health indicators like glycemic control, lipid metabolism, and inflammation—key determinants of healthy aging [83].

#### 2.8.2. Behavioral Analysis Using AI to Promote Healthy Aging

AI-driven behavioral analytics enable deeper insights into patterns of human behavior, motivation, and adherence to interventions designed to promote healthy aging. Machine learning algorithms and natural language processing (NLP) techniques applied to wearable sensors, mobile applications, and ambient sensors help identify behavioral patterns associated with positive or negative health outcomes, facilitating targeted and proactive interventions [72]. By continuously tracking activity levels, dietary habits, medication adherence, and emotional states, AI platforms can detect subtle deviations in behaviors predictive of health deterioration or reduced adherence. Predictive analytics and reinforcement learning techniques further assist in tailoring intervention strategies—such as personalized prompts, motivational messages, and adaptive goal setting—to promote sustained behavioral change and improve intervention adherence [32]. Furthermore, AI-driven emotional analytics, leveraging voice recognition, facial expression analysis, and NLP, facilitate proactive identification and intervention in mental health challenges common in aging populations, such as depression, anxiety, and social isolation. These insights enable timely psychological interventions, significantly enhancing overall well-being and quality of life in elderly individuals [72].

#### 2.8.3. Potential AI-Augmented Countermeasures to Social Isolation and Loneliness

The severe degradation of multiple aspects of general health occurring as a result of psychological stress is well documented [84] and is a major contributor to inflammaging [85]. Persons experiencing stress due to social isolation and loneliness are particularly vulnerable to dysregulation of inflammatory processes leading to cardiovascular disease [86]. Social isolation and loneliness significantly predict morbidity and mortality, particularly among the elderly [87,88]. Interestingly, loneliness and social isolation in veterans with wartime trauma are observed to correlate with telomere erosion—an objective indicator of biological age [89]—a finding with potentially enormous significance to long-term treatment for victims of wartime trauma. AI-assisted robotics and lifestyle interventions have potential for greatly improving stress-mediated contributors to adverse effects of aging, particularly among vulnerable populations, including war victims and the elderly, as described above in this section of the report.

#### 2.8.4. Examples of Successful AI-Driven Lifestyle Intervention Platforms

Numerous successful AI-driven platforms have demonstrated substantial impacts on healthspan and healthy aging through lifestyle interventions. For example, AI-powered applications such as Livongo and Omada Health have successfully used predictive analytics and personalized coaching to manage chronic conditions like diabetes and cardiovascular disease, leading to improved clinical outcomes, increased adherence to lifestyle modifications, and enhanced patient engagement [90]. Additionally, platforms such as Viome and DayTwo leverage microbiome analysis and AI-driven nutritional recommendations to optimize dietary strategies at an individualized level, resulting in measurable improvements in metabolic markers, weight management, and gastrointestinal health—key areas critical to healthspan extension [91]. Another prominent example, the WHOOP wearable platform, integrates AI analytics to deliver personalized recommendations on sleep, recovery, and physical performance, guiding individuals toward optimized lifestyle habits tailored to their physiological states. This approach has demonstrated effectiveness in enhancing overall physiological resilience, promoting sustained behavioral change, and improving quality-of-life metrics essential for healthy aging [92]. In summary, AI-driven lifestyle and behavioral interventions offer robust capabilities to precisely tailor recommendations and proactively encourage adherence, significantly advancing the effectiveness of personalized healthspan and longevity strategies.

It is also important to note gender differences in longevity research, as sex-specific variations influence aging biomarkers, drug responses, elderly care, and lifestyle interventions [93], reflecting Sydenham’s maxim that, “a man has the age of his arteries” [94].

### 2.9. Multi-Threat Medical Countermeasures (MTMC): Use of AI in Force Health Protection and Care for Victims of War

#### 2.9.1. Chronic Inflammatory Disorders: A Consequence of Modern Warfare

At the time of this writing, escalating military conflicts worldwide are subjecting increasingly large numbers of people to wartime injury and debilitation. Indeed, modern warfare has engendered both acute and chronic health effects in both military and civilian populations. The resulting burden on the healthcare infrastructure is enormous. For example, large-scale employment of chemical weapons, particularly sulfur mustard, by Iraq against Iranian soldiers and civilians, has resulted in over 34,000 chronically ill survivors of the exposures, which occurred during the Iran-Iraq War of the 1980s [95]. Significantly mustard-exposed persons suffered for decades from chronic inflammatory disorders—particularly lung, eye, and skin lesions as late complications in 34,000 Iranians with wartime exposure to this weapon, which was used extensively by Iraq as a combat force multiplier. The underlying pathogenesis of these disorders was complex and often difficult to treat long-term with single-site-acting small molecule drugs—although a major commonality was severely dysregulated inflammation and resulting tissue damage [92]. Within the domain of military medicine, this phenomenon served as an incentive to search for single drugs capable of counteracting multiple processes leading to inflammatory issue damage. This investigative and treatment-oriented paradigm, known as Multi-Threat Medical Countermeasures (MTMCs), evolved in recognition that almost all disorders known—even some such as post-traumatic stress syndrome (PTSD)—are driven to some extent by dysregulated inflammation. Indeed, there is no understating the significance of this recognition to members of the armed services, our brethren, who may have PTSD and CPTSD. Military veterans and first responders represent populations of high relevance in AI-driven aging and inflammation research due to their exposure to chronic stress, environmental insults, and traumatic injury. Increasing evidence indicates that post-traumatic stress disorder (PTSD) accelerates biological aging through mechanisms involving chronic inflammation, oxidative stress, telomere shortening, and mitochondrial dysfunction [96,97,98].

AI systems capable of analyzing large-scale biomarker and clinical datasets may offer improved stratification of such at-risk cohorts, guiding tailored interventions that mitigate aging-related decline. The applicability of AI-optimized anti-inflammatory or MTMC regimens to these populations offers a critical translational opportunity within the broader scope of healthspan extension.

#### 2.9.2. MTMC and Aging: Concept and Significance of Multi-Threat Medical Countermeasures (MTMC) in Aging

Inflammation is a cause or cofactor in most insults and illnesses and a common denominator among efficacious drugs and compounds for aging and chronic disease [99,100]. This includes a range of polypharmacy combinations involving a few key molecules shown to influence aging (Table 1). Any drug or compound that alleviates pathology resulting in cellular or tissue damage will likely affect inflammation and/or “inflammaging.” Almost all of the top ten prescribed drugs are primarily anti-inflammatory or exert pleiotropic, polypharmacy-like effects. The same is true for most compounds that have demonstrated anti-aging action or efficacy against chronic disease [101]. Much of this knowledge was assembled at the U.S. Army Medical Research Institute of Chemical Defense (USAMRICD) in the 1990s–2000s [102,103,104,105]. MTMC and checkpoint immunotherapy efficacy are linked by the clinically available inflammatory biomarker hs-CRP [101,106,107,108]. A better understanding of MTMC interactions with cancer-associated inflammation—and the three-piece puzzle of cellular receptors, soluble receptors, and ligands, as first presented in seminal work that now defines a new paradigm—can improve efficacy and reduce immunotherapy costs [109,110,111,112,113]. Notably, the chemical program previously worked with the Faber Institute to develop cancer chemotherapies. Colleagues at USAMRICD and Cowan, who developed the first experimental checkpoint drug at NIH in the 1970s, connected inflammation to primary data at the institute, formulating the Multi-Threat Medical Countermeasures (MTMC) hypothesis emphasizing broad-spectrum efficacy through synergistic polypharmacy [102,103,104,105]. This expanded to include lifestyle, diet, and supplements alongside pharmaceuticals, with inflammation as a central, though not exclusive, pathology and pharmacologic target [22,103,107,114]. Both the nerve agent soman and the first checkpoint therapy—a soluble Fc receptor (FcR)—demonstrated lethal toxicity at comparable molecular doses. Anaphylactoid reactions had also been reported in soman-poisoned animals by other investigators [104,111]. Inflammation associated with sulfur mustard, long assumed secondary to other biochemical pathways, was re-evaluated as a potential primary cause [62,63,64] Inflammation is now recognized as a key factor in inhalation, vesicating, and nerve agent toxicity. This body of work helped form the foundation for a billion-dollar effort at BARDA [115] to develop and repurpose drugs addressing common pathologies rather than specific illnesses or insults, with broad-spectrum “agnostic” MTMC. Multi-Threat Medical Countermeasures (MTMC) represent a sophisticated and integrated therapeutic strategy to simultaneously target multiple biological pathways involved in aging, disease, and environmental insult. These regimens combine pharmaceuticals, nutritional supplements, dietary interventions, and lifestyle modifications to address chronic inflammation, metabolic dysfunction, cellular senescence, and immune dysregulation—key mechanisms of aging [99].

#### 2.9.3. Role of AI in Optimizing MTMC Regimens

Artificial intelligence (AI) plays a critical role in optimizing MTMC interventions by systematically addressing complexities inherent in polypharmacy and multidimensional treatment protocols. Machine learning (ML) algorithms effectively analyze large-scale datasets to identify synergistic drug combinations, minimizing adverse interactions and amplifying therapeutic effects. AI-driven predictive analytics further personalize treatment regimens by integrating individual genomic, biochemical, clinical, and behavioral profiles, enabling precise customization of multi-threat interventions [51]. AI techniques, including deep learning and reinforcement learning, facilitate iterative optimization of MTMC protocols, dynamically adjusting therapeutic combinations and dosing regimens based on real-time patient monitoring data and predictive modeling outcomes. This iterative process ensures continuous improvement in regimen efficacy, patient adherence, and safety profiles, ultimately enhancing clinical outcomes related to longevity [34].

#### 2.9.4. AI-Enhanced MTMC Interventions: Targeting Chronic Inflammation and Metabolic Health

Recent research has demonstrated significant success in applying AI-driven MTMC interventions targeting chronic inflammation and metabolic dysfunction. For example, combinatorial therapies involving senolytic agents (such as dasatinib and quercetin), NAD+ precursors (nicotinamide riboside or nicotinamide mononucleotide), anti-inflammatory dietary supplements (curcumin, omega-3 fatty acids, and resveratrol), and lifestyle interventions (caloric restriction mimetics and intermittent fasting) have shown enhanced efficacy in animal and preliminary human studies when optimized by AI [116]. AI algorithms have successfully predicted patient-specific inflammatory and metabolic responses to these combinatorial interventions, identifying optimal therapeutic dosages, timing, and sequencing to maximize anti-inflammatory and anti-senescent effects. This personalized approach significantly improves metabolic markers such as glucose metabolism, lipid profiles, oxidative stress indicators, and inflammatory biomarkers (C-reactive protein, IL-6, TNF-α), which strongly correlate with healthspan and longevity outcomes [100,116].

#### 2.9.5. Case Studies and Evidence of AI-Optimized MTMC Success

Emerging clinical evidence highlights the substantial potential of AI-driven MTMC regimens. For instance, pilot studies using AI-designed polypharmacy protocols—including rapamycin analogs, senolytic cocktails, mitochondrial enhancers, and targeted nutraceutical combinations—demonstrated measurable improvements in biomarkers indicative of reduced inflammation, cellular rejuvenation, and metabolic optimization [117]. Such AI-enhanced protocols substantially reduced inflammatory cytokines, improved mitochondrial function, enhanced physical performance metrics, and demonstrated favorable safety profiles compared to conventional treatment approaches. Another notable study utilized AI modeling to personalize combinations of lifestyle modification (dietary fasting, exercise protocols), metformin, and NAD+ precursors, demonstrating significant improvements in insulin sensitivity, inflammatory markers, and functional aging parameters in human subjects, validating the practical feasibility and clinical value of AI-optimized MTMC strategies [116].

#### 2.9.6. Future Directions and Clinical Implications

The continued development and refinement of AI-enhanced MTMC interventions hold significant promise for future radical longevity efforts. Strategic directions should emphasize robust clinical validation, longitudinal outcome tracking, and regulatory approval pathways for AI-driven polypharmacy interventions. Furthermore, advancing computational models to enhance precision in patient-specific predictions and to dynamically adapt interventions throughout the aging trajectory will substantially increase clinical effectiveness and real-world applicability. In conclusion, AI-optimized MTMC regimens represent a highly promising, effective, and scalable approach for combating chronic inflammation, improving metabolic health, and achieving meaningful improvements in healthspan and radical longevity.

## 3. Discussion

### 3.1. Ethical and Societal Considerations in AI-Driven Longevity Research

The integration of artificial intelligence (AI) into longevity research and practice offers substantial promise but simultaneously raises significant ethical and societal considerations. Foremost among these concerns are issues related to equity, accessibility, privacy, autonomy, and potential societal consequences of substantially increased human lifespans [118]. Equitable access to AI-enhanced longevity technologies represents a profound ethical challenge. There is a substantial risk that advanced longevity interventions—driven by personalized, AI-informed strategies—could exacerbate existing health disparities, disproportionately benefiting affluent populations while limiting accessibility to economically disadvantaged communities [2]. Addressing these disparities requires proactive, inclusive policies aimed at ensuring the broad, equitable distribution and affordability of emerging AI-driven healthspan interventions. Privacy and data security issues also arise from the extensive data collection inherent to AI-driven longevity interventions. AI analytics, reliant on comprehensive and sensitive personal data—including genomic, biometric, behavioral, and environmental information—pose risks regarding data misuse or unauthorized disclosure, thereby necessitating robust, transparent, and ethically governed data management strategies [119]. Moreover, the pursuit of radical longevity raises broader societal questions surrounding population growth, resource allocation, intergenerational equity, and potential impacts on societal structures such as retirement, employment, and healthcare provision. These implications necessitate careful deliberation among policymakers, ethicists, researchers, and the public to navigate the complex societal transformations that extended lifespans could engender [118].

### 3.2. Challenges, Limitations, and Future Directions

Despite substantial advancements, integrating AI into longevity research faces notable challenges and limitations. A key limitation involves data quality, standardization, and interoperability across diverse datasets, particularly multi-omics, clinical, and behavioral data, which vary significantly in format, quality, and collection methodologies. Resolving these issues requires standardized data collection frameworks, consistent analytic methodologies, and rigorous data validation processes [34]. Another significant challenge pertains to the explainability and interpretability of AI algorithms, particularly deep learning models, which often function as “black boxes.” The opacity of AI-driven insights complicates clinical trust, regulatory acceptance, and patient comprehension, thereby emphasizing the need for developing transparent, explainable AI models suitable for clinical application in longevity interventions [120]. Additionally, ethical, regulatory, and logistical barriers exist for the clinical validation and approval of AI-driven longevity therapies, particularly regarding precision-medicine approaches and personalized interventions. Developing frameworks that adequately evaluate AI-driven predictive accuracy, safety, efficacy, and long-term outcomes is essential for translating AI technologies effectively into clinical longevity practice [10,49]. Future directions for AI-driven longevity research include increased emphasis on explainable AI, real-world validation of predictive models, and integration of AI-driven platforms within healthcare delivery systems. Additionally, promoting interdisciplinary collaboration among AI scientists, biomedical researchers, clinicians, ethicists, and policymakers is crucial for addressing multifaceted challenges associated with AI-enabled longevity interventions.

### 3.3. Strategic Recommendations for Integrating AI into Longevity Practices

To maximize AI’s transformative potential in longevity medicine, several strategic recommendations are essential:

Interdisciplinary Collaboration: Promote sustained interdisciplinary partnerships among computational experts, clinicians, gerontologists, ethicists, policymakers, and healthcare administrators to comprehensively address scientific, ethical, and societal dimensions of AI-driven longevity innovations.

Data Standards and Transparency: Establish standardized, interoperable data frameworks to enhance AI model training, validation, and reproducibility across research institutions, clinical settings, and industry partnerships. Data transparency, privacy safeguards, and secure data-sharing protocols should accompany these standards.

Explainability and Clinical Trust: Develop explainable and transparent AI methodologies suitable for clinical decision-making. Clear communication of AI-driven recommendations to healthcare providers and patients can improve acceptance, trust, and adherence to personalized longevity strategies.

Equitable Access and Inclusivity: Formulate policies and funding structures explicitly designed to ensure equitable access to AI-driven longevity interventions across diverse socio-economic and demographic populations, addressing and reducing healthcare disparities.

Regulatory Frameworks: Advocate for adaptable, evidence-based regulatory guidelines specifically tailored to AI-driven longevity technologies, facilitating timely validation, approval, and implementation of novel interventions while safeguarding patient safety and privacy.

Taking these considerations into account will enhance optimization of AI’s full potential in longevity science necessitates careful attention to ethical and societal considerations, targeted strategies to overcome existing limitations, and thoughtful integration of AI-driven solutions into clinical and societal frameworks. By addressing these challenges proactively, AI-driven longevity research promises to fundamentally transform human health, well-being, and longevity.

## 4. Conclusions

Artificial intelligence represents a powerful catalyst for revolutionizing longevity science, fundamentally enhancing our ability to understand, predict, and intervene in aging processes. The integration of machine learning, deep learning, natural language processing, robotics, and advanced analytics significantly accelerates biomarker discovery, precision medicine, personalized lifestyle interventions, drug development, and clinical research efficiency, offering unprecedented potential for extending healthspan and achieving radical longevity. However, fully realizing this potential necessitates careful navigation of critical ethical and societal considerations—including equity, accessibility, privacy, and broader socio-economic implications associated with significantly extended lifespans.

Moreover, in order to fully optimize the value of AI and other information technologies, it is important to understand their limitations in improving the human condition.

Most notably, despite rapidly evolving improvements in AI and other information technologies, the impact on disease mortality has slowed due to complex, multifactorial causes, including lifestyle, access disparities, and chronic inflammatory disorders [100]. This highlights the importance of AI to biomedical science, not as a panacea, but as a complementary tool.

Addressing these challenges requires proactive interdisciplinary collaboration, transparent and explainable AI methodologies, robust data governance, equitable distribution strategies, and adaptable regulatory frameworks tailored to the unique needs of AI-driven longevity research and practice. Essential future pathways must emphasize interdisciplinary partnerships, the validation of AI-driven interventions in real-world clinical settings, and the development of inclusive policies designed to democratize access to longevity innovations. By thoughtfully integrating AI into the broader framework of healthcare and societal practices, longevity science can move closer to fulfilling its promise of substantially improved human health, well-being, and quality of life. Finally, this report further highlights hemolytic disorders and cardiovascular senescence as model systems to demonstrate how AI can be applied to complex, multifactorial aging syndromes through integrated, broad-spectrum interventions.

## Figures and Tables

**Table 1:** MTMC anti-inflammaging drug and compounds.

Drug/Supplement	Anti-Inflammatory	Action/Mechanisms
Metformin	Yes	AMPK activation, insulin sensitization, gut microbiome modulation; COVID-19 benefit
Hydrocortisone	Yes (Glucocorticoid)	Suppresses innate immune response
Testosterone	Yes (context-dependent)	Neuroendocrine modulation; cognitive support, mood regulation
Diclofenac	Yes (NSAID)	COX inhibition; pain reduction
Dasatinib	Yes (Senolytic)	Tyrosine kinase inhibitor
DHA (Docosahexaenoic acid)	Yes (Omega-3)	Brain protection; mitochondrial support
Vitamin D3 (Cholecalciferol)	Yes	Immune modulation; bone health
Estradiol	Yes	Hormonal balance; vascular protection; neuroprotection
Mecamylamine	Yes	Cholinergic anti-inflammatory reflex; nicotinic receptor blockade
Nicotine	Yes	Alpha-7 nicotinic receptor agonist; neurostimulation; cognitive enhancement
Quercetin	Yes	Antioxidant; senolytic; immune regulation
Resveratrol	Yes	SIRT1 activation; mitochondrial and cardiovascular support
Sirolimus (Rapamycin)	Yes	mTOR inhibition; cognitive improvement; tumor suppression; lupus; GVHD; COVID-19
Curcumin	Yes	COX/LOX inhibition; BDNF enhancer; antioxidant
Deprenyl (Selegiline)	Yes (indirect)	MAO-B inhibition; dopaminergic and cognitive support
GLP-1 Agonists	Yes	NF-κB and TLR modulation; metabolic regulation; weight loss; broad MTMC effects
Melatonin	Yes	Antioxidant; circadian rhythm regulation; neuroprotection
N-Acetylcysteine (NAC)	Yes	Glutathione precursor; antioxidant; respiratory and neurological support
Berberine	Yes	AMPK activation; anti-diabetic; metabolic and cardiovascular benefits
Pterostilbene	Yes	SIRT1 activation; antioxidant; improved bioavailability over resveratrol

## Data Availability

Not applicable.
